# Performance of Natural Language Processing for Information Extraction From Electronic Health Records Within Cancer: Systematic Review

**DOI:** 10.2196/68707

**Published:** 2025-09-12

**Authors:** Simon Dahl, Martin Bøgsted, Tomer Sagi, Charles Vesteghem

**Affiliations:** 1 Center for Clinical Data Science, Department of Clinical Medicine, Aalborg University, Selma Lagerløfs Vej 249, Gistrup, 9260, Denmark, +45 99407244; 2 Center for Clinical Data Science, Research, Education and Innovation, Aalborg University Hospital, Aalborg, Denmark; 3 Clinical Cancer Research Centre, Department of Clinical Medicine, Aalborg University Hospital, Aalborg, Denmark; 4 Department of Computer Science, Aalborg University, Aalborg, Denmark

**Keywords:** natural language processing, information extraction, clinical textual data, performance, *F*_1_-score, review, rule-based solutions, traditional machine learning, neural network, bidirectional transformer

## Abstract

**Background:**

Over the last decade, natural language processing (NLP) has provided various solutions for information extraction (IE) from textual clinical data. In recent years, the use of NLP in cancer research has gained considerable attention, with numerous studies exploring the effectiveness of various NLP techniques for identifying and extracting cancer-related entities from clinical text data.

**Objective:**

We aimed to summarize the performance differences between various NLP models for IE within the context of cancer to provide an overview of the relative performance of existing models.

**Methods:**

This systematic literature review was conducted using 3 databases (PubMed, Scopus, and Web of Science) to search for articles extracting cancer-related entities from clinical texts. In total, 33 articles were eligible for inclusion. We extracted NLP models and their performance by *F*_1_-scores. Each model was categorized into the following categories: rule-based, traditional machine learning, conditional random field-based, neural network, and bidirectional transformer (BT). The average of the performance difference for each combination of categorizations was calculated across all articles.

**Results:**

The articles covered various scenarios, with the best performance for each article ranging from 0.355 to 0.985 in *F*_1_-score. Examining the overall relative performances, the BT category outperformed every other category (average *F*_1_-score between 0.2335 and 0.0439). The percentage of articles on implementing BTs has increased over the years.

**Conclusions:**

NLP has demonstrated the ability to identify and extract cancer-related entities from unstructured textual data. Generally, more advanced models outperform less advanced ones. The BT category performed the best.

## Introduction

Electronic health records (EHRs) are increasingly being adopted by health care providers worldwide, as they offer numerous benefits [1]. This has led to an increase in the quantity of data stored in EHRs, consisting of both structured and unstructured data (eg, text, images, and time series). Unstructured textual data from discharge summaries, radiology reports, clinical notes, and patient histories provide valuable information about patients that may not be captured by structured data alone [2]. The extraction of clinical parameters from unstructured textual data, also known as information extraction (IE), has proven to be valuable in health care, such as in clinical research (eg, epidemiology) and decision support systems [3,4].

However, working with unstructured textual data presents several challenges to health care providers and researchers. The volume of free text makes manual extraction and analysis time-consuming and resource-heavy, thereby limiting their utility and requiring automated solutions. Moreover, the lack of standardization and consistency in formatting and terminology makes it difficult to accurately identify and extract the relevant information in an automated manner. Furthermore, free text is prone to spelling errors, resulting in inaccurate or harder-to-find patient information for methods that rely on keyword extraction or other exact-match search techniques.

Natural language processing (NLP) techniques are well suited for extracting information from free text because of their ability to process, comprehend, and generate human language in a manner that allows for automatic extraction of structured information from free text. In recent years, NLP has gained considerable attention, with numerous studies exploring the effectiveness of various NLP techniques, notably for identifying and extracting cancer-related entities, such as smoking history [2], toxicities [5], and Gleason scores [6], which are only recorded as free text in clinical notes. These techniques are known as named entity recognition, or more generally, IE [7-9].

A variety of techniques and pipelines have been developed for IE from medical free texts, ranging from simple rule-based solutions to advanced machine learning approaches. Rule-based solutions allow domain experts to define a set of linguistic rules and patterns to be implemented to identify and extract relevant information from clinical notes and medical free-form texts. Studies have shown that rule-based approaches can outperform machine-learning models [10-13]. However, rule-based approaches are custom-made for specific datasets and use cases, require manual specification of rules by medical experts, and are therefore difficult to generalize [3]. Moreover, machine learning models allow for multiple methodologies and applications for different IE problems, solved by training specific traditional machine learning models such as support vector machines, decision trees, and neural networks (NNs). Recently, bidirectional transformers (BTs) such as large language models (LLM) have been identified as a possible tool for IE because of their strengths in pattern recognition, text summarization, and generation [14]. LLMs allow for pretraining on large text corpora, which enables them to learn linguistic patterns applicable to different IE tasks. Furthermore, LLMs show promising results for specific tasks because of the domain-specific fine-tuning of pretrained models [15].

Over the years, numerous models have been developed for various IE tasks, but their relative performance across different datasets remains largely unknown. Both rule-based and machine learning approaches often exhibit limited generalizability between tasks, and their results are frequently inconsistent when applied to different datasets. This inconsistency highlights the need for studies that investigate and compare the relative performance of these models.

To the best of our knowledge, no review has been conducted that summarizes the differences in the performance of various types of NLP models for IE within the context of cancer. This review provides an overview of the various NLP methods used for IE and compares them in terms of their performance.

## Methods

This systematic review followed the Preferred Reporting Items for Systematic Reviews and Meta-Analyses (PRISMA) guidelines.

We searched 3 databases—PubMed, Scopus, and Web of Science—for relevant literature published between January 1, 2014, and April 19, 2024 (Multimedia Appendix 1). The following search criteria were used for the titles and abstracts:

(“information extraction” OR “natural language processing” OR “nlp”) AND (EHR OR notes OR reports) AND (cancer OR tumor OR oncology)

The inclusion criterion for articles was the application of 2 or more NLP models to extract identical cancer-related entities from the same unstructured medical text in EHR. Articles were excluded during title and abstract screening if they were as follows: (1) reviews; (2) articles that do not compare 2 or more NLP models; (3) articles whose purpose is not to extract information from free text from EHR; and (4) articles whose purpose is not to develop an NLP cancer-related application.

For further exclusion during the full-text screening, articles were excluded if they were defined as follows: (1) abstract only; (2) text classification without cancer entity extraction; (3) results within the article were not compatible; (4) no NLP application development; (5) not related to cancer IE from EHR; and (6) no comparison with other NLP methods within the article.

Using the exclusion criteria, one author (SCD) performed 2 rounds of article selection: title and abstract screening, followed by a full-text review. A second reviewer (CV) was consulted for unclear cases during the screening.

Data from each of the included articles were extracted by 2 authors (SCD and CV). Both authors independently categorized the NLP models and extracted their performance metrics. Any discrepancies in categorization were resolved through consensus guided by consideration of the primary architectural components of the model. Each model was categorized into the following groups: rule-based, traditional machine learning (ML), conditional random field (CRF)–based, NN, and BT.

The rule-based category includes IE models that use regular expressions (Regex), keywords, and dictionary matching. The CRF-based category includes linear CRF, except bidirectional long short-term memory-CRF, which is in the NN category. The NN category includes NNs, except for BTs, that belong to the BT category. Ensemble models are categorized as the most advanced part of the ensemble. For example, a rule-based model combined with a BT is categorized as a BT model (see Table 1). For articles that included both strict and relaxed keyword matching, the strict *F*_1_-scores were extracted as the performance metric. For articles presenting both macro- and micro-averaged *F*_1_-scores, macro-averaged *F*_1_-scores were extracted.

**Table 1.** Method categorization of models.

Category	Included models	Articles using category	Total number of models implemented
Rule-based	Regular expressions keyword, term, and dictionary matching	[10-13,16-22] (n=11)	12
CRF^a^-based	Linear CRF CRF + Rule-based	[10,12,16-18,20,23,24] (n=8)	26
Bidirectional transformer	BERT^b^ BlueBERT BioBERT CharBERT Character-BERT CancerBERT RoBERTa MBERT (multilingual BERT) BETO XLM-R ClinicalBERT XLNet Bidirectional Transformer + Rule-based Bidirectional Transformer + BiLSTM^c^-CRF	[11,15-17,21,23-33] (n=16)	60
Neural network	BiGRU BiRNN CNN LSTM BiLSTM-CRF RNN MLP, HAN, SLA, CNN + Rule-based	[15-20,24-26,28-43] (n=25)	83
Traditional machine learning	SVM^d^ Random forest Naïve Bayes Extreme Gradient Boosting AdaBoost	[13,22,27,30,34-43] (n=14)	39

a CRF: conditional random field.

b BERT: Bidirectional Encoder Representations from Transformers.

c BiLSTM: bidirectional long short-term memory.

d SVM: support vector machine.

To calculate the performance differences for all categories across the included articles, the following steps were executed for all categories.

The best-performing model for each category within each article was selected. The best-performing model within category *c* for article *a* is given by ${max}_{c,a}$:


$${max}_{c,a}=\max(P_{{c,m}^{1}},P_{{c,m}^{2}},\ldots ,P_{c,m^{n}})$$

where *P* is the *F*_1_ performance score of method *m* within category *c. n* is the number of methods within category *c*.

Having the best-performing categories within an article allows for calculation of the category difference for each combination of categories. Category differences for categories *c^1^* and *c^2^* within article *a* are given as ${category\_diff}_{c^{1},c^{2},a}$:


$${category\_diff}_{c^{1},c^{2},a}={max}_{c^{1},a}-{max}_{c^{2},a}$$

where ${max}_{c,a}$ is the best performing model of category *c* in article *a*.

All performance differences of the same combination of categories were averaged across all the articles. The average of the category difference for all articles with combination *c^1^* and *c^2^* is given as ${performance\_difference}_{c1,c2}$ :


$$performance\_difference_{c1,c2}=\frac{category\_diff_{c^{1},c^{2},a^{1}}+category\_diff_{c^{1},c^{2},a^{2}}+\cdots +category\_diff_{c^{1},c^{2},a^{n}}}{n}$$

where ${category\_diff}_{c^{1},c^{2},a}$ is the category difference between *c^1^* and *c^2^* in article *a. n* is the number of articles with a specific category combination.

Statistical significance between categories for each category combination was determined using a *t* test (*P*<.05).

## Results

### Overview

**Figure 1.**
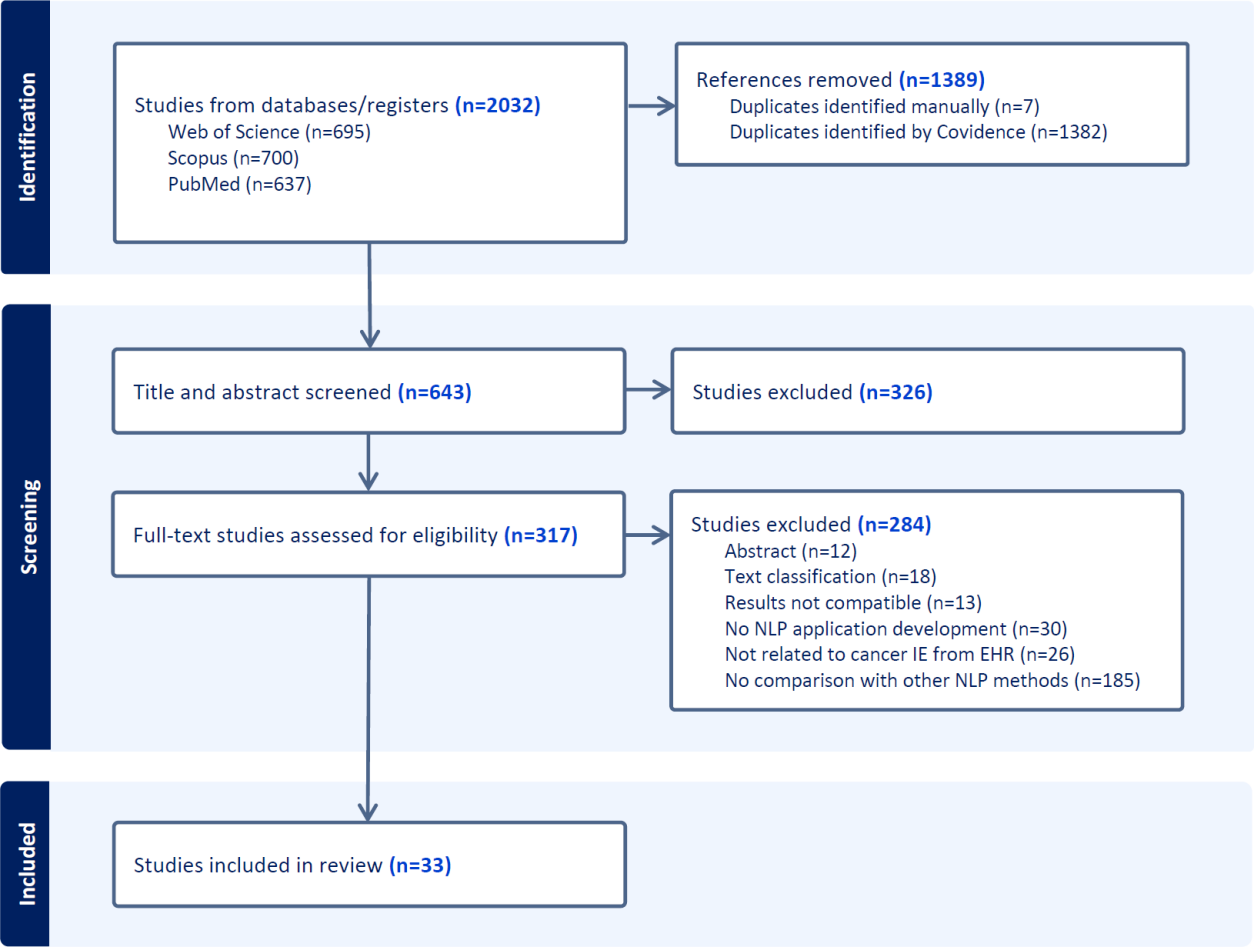
The PRISMA (Preferred Reporting Items for Systematic Reviews and Meta-Analyses) flowchart for article selection.

The article selection process is detailed in a PRISMA flowchart, shown in Figure 1. A total of 2032 articles were identified through searches in Web of Science, Scopus, and PubMed.

In total, 33 articles were included in this review. The articles were published between 2018 and 2024. They compared at least 2 NLP models for cancer-related IE from unstructured medical texts in EHRs. The articles contained a total of 220 implementations of NLP models. Selecting only the best-performing models within each category of each article summarizes 74 implementations.

### Models

We categorized each NLP model as rule-based, CRF-based, BT, NN, and ML. Table 1 shows how each model was categorized and the articles in which the categories are contained.

Table 1 shows the categorization of the models, which articles contain the specific categories, and the total number of implemented models within each category.

**Figure 2.**
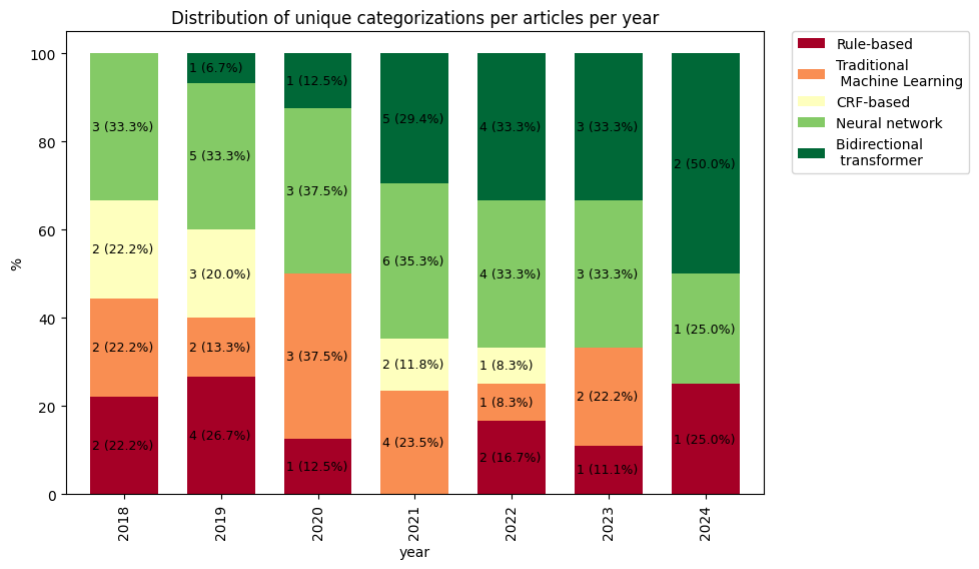
Distribution of unique categorizations per article per year. CRF: conditional random field.

The most frequently used category was NN, with 25 occurrences, followed by BT and ML with 16 and 11 occurrences, respectively. The most frequently implemented category was NN, with 83 implementations. The distribution of unique categorizations per year shows the variety of models that have been used throughout the years (see Figure 2). Notably, the percentage of articles on the implementation of BTs has increased over the years.

### Performance

The performance varied a lot according to specific use cases. Inspecting the best-performing models of the specific articles shows that a total of 5 rule-based models performed best in their articles, with *F*_1_-scores in the range of 0.73-0.887 (see Table 2). ML did not perform best in any article despite being compared in 14 articles with a total of 39 different model implementations, and neither did CRF-based. In total, 14 articles showed that NN performed the best, with *F*_1_-scores ranging from 0.3539 to 0.972. BT performed the best in 13 articles, with *F*_1_-scores ranging from 0.6023 to 0.97. Looking at the raw *F*_1_-scores, more advanced models outperformed less advanced ones.

**Table 2.** Article overview.

Article	Year	Title	Number of tested models (best performing *F*_1_-score within category)
AAlAbdulsalam et al [10]	2018	Automated extraction and classification of cancer stage mentions from unstructured text fields in a central cancer registry	Rule-based=1 (0.887)^a^; CRF^b^-based=1 (0.882)
Alawad et al [36]	2018	Coarse-to-fine multi-task training of convolutional neural networks for automated information extraction from cancer pathology reports	ML^c^=1 (0.626); NN^d^=2 (0.752)^a^
Miao et al [20]	2018	Extraction of BI-RADS findings from breast ultrasound reports in Chinese using deep learning approaches	Rule-based=1 (0.848); CRF-based=1 (0.881); NN=2 (0.904)^a^
Qiu et al [35]	2018	Deep learning for automated extraction of primary sites from cancer pathology reports	ML=3 (0.640); NN=3 (0.701)^a^
Chen et al [12]	2019	Using natural language processing to extract clinically useful information from Chinese electronic medical records	Rule-based=1 (0.83)^a^; CRF=1 (0.8)
Coquet et al [19]	2019	Comparison of orthogonal NLP methods for clinical phenotyping and assessment of bone scan utilization among prostate cancer patients	Rule-based=1 (0.897); NN=3 (0.918)^a^
Dubey et al [37]	2019	Inverse regression for extraction of tumor site from cancer pathology reports	ML=5 (0.759)^a^; NN=2 (0.701)
Kim et al [18]	2019	A study of medical problem extraction for better disease management	Rule-based=2 (0.883); CRF-based=4 (0.926); NN=5 (0.929)^a^
Thompson et al [34]	2019	Relevant word order vectorization for improved natural language processing in electronic health records	ML=7 (0.788); NN=7 (0.858)^a^
Zhang et al [17]	2019	Extracting comprehensive clinical information for breast cancer using deep learning methods	Rule-based=1 (0.484); NN=1 (0.887); CRF-based=1 (0.837); BT^e^=1 (0.935)^a^
Alawad et al [40]	2020	Automatic extraction of cancer registry reportable information from free-text pathology reports using multitask convolutional neural networks	ML=2 (0.615); NN=3 (0.752)^a^
Odisho et al [28]	2020	Natural language processing systems for pathology parsing in limited data environments with uncertainty estimation	ML=4 (0.948); NN=2 (0.972)^a^
Osborne et al [11]	2020	Identification of cancer entities in clinical text combining transformers with dictionary features	Rule-based=1 (0.73)^a^; BT=7 (0.7)
Wu et al [38]	2020	Structured information extraction of pathology reports with attention-based graph convolutional network	ML=1 (0.74); NN=6 (0.803)^a^
Hu et al [28]	2021	Automatic extraction of lung cancer staging information from computed tomography reports: deep learning approach	NN=2 (0.773); BT=1 (0.81)^a^
Liu et al [24]	2021	Use of BERT (Bidirectional Encoder Representations from Transformers)-based deep learning method for extracting evidences in Chinese radiology reports: development of a computer-aided liver cancer diagnosis framework	CRF-based=1 (0.729); NN=1 (0.832); BT=1 (0.857)^a^
López-García et al [23]	2021	Detection of tumor morphology mentions in clinical reports in Spanish using transformers	CRF-based=1 (0.794); BT=18 (0.89)^a^
Lu et al [27]	2021	Natural language processing and machine learning methods to characterize unstructured patient-reported outcomes: validation study	ML=2 (0.365); BT=1 (0.602)^a^
Park et al [43]	2021	Improving natural language information extraction from cancer pathology reports using transfer learning and zero-shot string similarity	ML=4 (0.484); NN=5 (0.502)^a^
Rios et al [42]	2021	Assigning ICD-O-3 codes to pathology reports using neural multi-task training with hierarchical regularization	ML=3 (0.276); NN=12 (0.355)^a^
Wu et al [41]	2021	BioIE: biomedical information extraction with multi-head attention enhanced graph convolutional network	ML=1 (0.444); NN=5 (0.613)^a^
Yu et al [26]	2021	A study of social and behavioral determinants of health in lung cancer patients using transformers-based natural language processing models	BT=4 (0.879)^a^; NN=2 (0.844)
Bozkurt et al [13]	2022	Expanding the secondary use of prostate cancer real world data: automated classifiers for clinical and pathological stage	Rule-based=1 (0.87)^a^; ML=1 (0.723)
Fang et al [16]	2022	Extracting clinical named entity for pituitary adenomas from Chinese electronic medical records	Rule-based=1 (0.431); CRF-based=16 (0.904); NN=1 (0.899); BT=1 (0.913)^a^
Hu et al [29]	2022	Using natural language processing and machine learning to preoperatively predict lymph node metastasis for non-small cell lung cancer with electronic medical records: development and validation study	NN=1 (0.701); BT=2 (0.948)^a^
Pabón et al [25]	2022	Negation and uncertainty detection in clinical texts written in Spanish: a deep learning-based approach	NN=2 (0.788); BT=1 (0.823)^a^
Zhou et al [15]	2022	CancerBERT: a cancer domain-specific language model for extracting breast cancer phenotypes from electronic health records	NN=1 (0.834); BT=8 (0.876)^a^
Ansoborlo et al [22]	2023	Prescreening in oncology trials using medical records. Natural language processing applied on lung cancer multidisciplinary team meeting reports	Rule-based=1 (0.932)^a^; ML=1 (0.68)
Rohanian et al [32]	2023	Using bottleneck adapters to identify cancer in clinical notes under low-resource constraints	NN=3 (0.83); BT=8 (0.97)^a^
Seong et al [31]	2023	Deep learning approach to detection of colonoscopic information from unstructured reports	NN=3 (0.985)^a^; BT=2 (0.982)
Zitu et al [30]	2023	Generalizability of machine learning methods in detecting adverse drug events from clinical narratives in electronic medical records	ML=1 (0.69); NN=2 (0.763); BT=2 (0.778)^a^
Martín-Noguerol et al [33]	2024	Natural language processing deep learning models for the differential between high-grade gliomas and metastasis: what if the key is how we report them?	NN=3 (0.872)^a^; BT=1 (0.766)
Hu et al [21]	2024	Zero-shot information extraction from radiological reports using ChatGPT	Rule-based=1 (0.926); BT=2 (0.957)^a^

a This is the best *F*_1_-score for the article.

b CRF: conditional random field.

c ML: traditional machine learning.

d NN: neural network.

e BT: bidirectional transformer.

Table 2 shows each article and the number of models in each category within the article. Parentheses show the best *F*_1_-score for each category. The best *F*_1_-score for each article is marked by a footnote.

**Figure 3.**
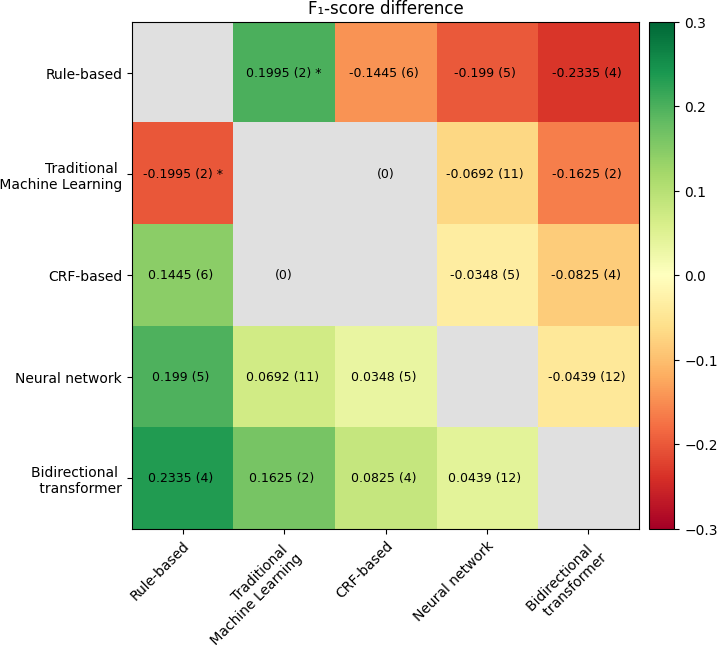
Illustration of the average *F*_1_-score performance differences for all combinations of the best model within each category. The parentheses indicate the number of comparisons between the categories. CRF: conditional random field. *P<.05.

Some variations between the average *F*_1_-score performance differences were observed (see Figure 3).

Our results show that more advanced models outperform less advanced ones. The largest difference between the category performance *F*_1_-scores was observed between the BT category and the rule-based category. BT models were compared with rule-based models in 4 studies, yielding an average performance difference of 0.2335 in terms of *F*_1_-score. BT was the best-performing category. NN outperformed CRF-based, ML, and rule-based models, while CRF-based outperformed rule-based models, and rule-based outperformed ML models. The only statistically significant difference between categories is observed when comparing rule-based and ML; see Multimedia Appendix 2 for *P* values.

## Discussion

### Principal Findings

This study provides an overview of the models used for IE in cancer and their performance in terms of the *F*_1_-score. By including only articles with 2 or more NLP models for IE, we were able to evaluate the relative performance of each NLP within categories: rule-based, CRF-based, BT, NN, and ML.

The search string for this review combined keywords for techniques (IE and NLP), data sources (EHR, notes, reports), and the domain (cancer, tumor, and oncology) using Boolean operators to limit irrelevant results. The initial yield of 2032 articles suggests a reasonable balance, considering the stringent inclusion criteria. The “AND” clauses effectively limit the search while still including the relevant articles for the screening process. Although our search strategy included articles published from 01/01/2014, no articles prior to 2018 were included in the analysis. The reason for this discrepancy is not addressed within the scope of this review, which focused on quantifying performance differences between our categories. Notably, the most frequent reason for full-text exclusion was “No comparison with other NLP methods within the article (185 articles).” Arguing for common benchmark testing of the implemented NLP models.

Without considering a dataset or specific extraction entities, our results show that BT is the best performing category, followed by NN, CRF-based, rule-based, and ML in written order. We observed an increasing number of transformer-based models developed in recent years, with promising results. Our results highlight a pivotal moment in which BTs, such as language models, are on the verge of demonstrating their full potential in IE. Although transformers [44] and BERT [45] were introduced in 2017 and 2018, respectively, our literature review includes no articles using these technologies until 2019. This delay in time may reflect the time required for these models to become integrated into clinical research workflows. Surprisingly, rule-based solutions perform better than machine learning [13,22]. One explanation could be that rule-based solutions allow for the implementation of expert knowledge. The lowest-performing articles in terms of *F*_1_-score do not aim to show the best possible method for extraction, but rather how *F*_1_-scores increase using hierarchical regularization when extracting ICD-O-3 codes [42]. Similarly, the study of Park et al [43] aims to show how to increase the *F*_1_-score, using transfer learning and zero-shot string similarity, when the number of annotated pathology reports is limited.

Multiple reviews have been conducted within the scope of NLP in a clinical context with different aims. The review by Kreimeyer et al [46] aims to identify NLP systems capable of processing clinical free text and generating structured output, thereby compiling a list of NLP solutions in use. The review by Datta et al [47] defines relevant linguistic terms by organizing unstructured clinical text related to cancer into structured data using frame semantics. The review by Bilal et al [48] examines the current state-of-the-art literature on NLP applications in analyzing EHRs and clinical notes for cancer research, quantifying the number of studies for each cancer type and outlining the research challenges and future directions for NLP when analyzing EHRs and clinical notes in cancer research. However, no review has been conducted comparing the performance of NLP models for IE of cancer-related entities from clinical text, a gap relevant to clinical informatics and crucial for improving the accuracy of cancer-related data IE within EHRs. This is the first review to summarize and compare the performance of NLP models for IE of different cancer entities from unstructured text, offering insights for clinical researchers focused on leveraging EHR data for cancer care and research.

### Strengths

One strength of our study was its ability to overcome the challenge of comparing low-performing models. By including only articles with 2 or more categories, we can determine the relative performance for each paper while neglecting low-performing models from papers that do not aim to beat state-of-the-art *F*_1_-score. Our review shows how models can be categorized and how the categorizations perform compared to each other through different datasets and extraction entities. The performance differences observed in our included articles highlight the importance of selecting the appropriate NLP model for each health care application. Our categorizations allow all models to be included, even ensemble and hybrid models. Furthermore, our performance calculation uses the best-performing model for each category reported within each included article. This approach allows for the addition of multiple new categories to support the desired level of model performance granularity.

### Limitations

A categorization strategy was required to categorize all models. Most models assign into well-defined and distinct categories. However, some could be assigned to multiple categories, notably bidirectional long short-term memory-CRF models. To present intelligible results, the number of categories had to be kept relatively low, neglecting model specificities. Increasing the number of categories would reduce the number of models in that category, making the results too anecdotal. Decreasing the number of categories would increase the number of times each categorization was compared, making the averaged *F*_1_-scores less distinctive. Ideally, we would have wished for multiple studies implementing the same set of models and categorizations to avoid certain categorizations not being compared with every other category and to avoid certain combinations of categorizations occurring only once.

We selected the *F*_1_-score as a metric for performance; precision or recall could also be used. However, extracting specific numbers from the confusion matrix can provide deeper insights. The included studies reported *F*_1_-scores as a measure of performance. Although this is a practical method to generate 1 performance metric, its use has some limitations. In medical IE, one could argue that false negatives are worse than false positives, potentially leading to missed diagnoses or inappropriate treatment decisions, which is not considered in the *F*_1_-score. While metrics such as AUC-ROC, precision-recall tradeoff, or specificity offer complementary insights, their calculation was limited by the inconsistent reporting of the necessary data. Furthermore, given the sensitive nature of EHR data and the need for clinical trust, future research should also prioritize evaluating the interpretability of IE models alongside traditional performance measures to allow clinicians to understand how cancer-related entities are being extracted and validated from EHR data.

Furthermore, our included studies neglected to address the handling of negation and spelling errors. Giorgia et al [49] showed that negations account for 66% of the errors. Another study stated that BERT fails completely to show a generalizable understanding of negation, raising questions about the aptitude of language models to learn this type of meaning [50]. In this study, BTs performed well; one could wish for a general approach to analyze the errors of each model instead of the general performance derived from the confusion matrix. Negation errors pose a significant challenge in EHR data and are critical in oncology, as a misidentified negated symptom or finding can alter clinical interpretation, treatment planning, and patient care.

### Perspectives

The field of IE has evolved rapidly, and models, such as LLMs, have been successfully applied in the context of cancer IE, both in terms of model performance and operational efficiency [51]. LLM could allow for enhanced transferability and utility for different IE tasks on unstructured textual data. Using LLMs for IE on unstructured textual data seems feasible because of the variety of available pretrained models in different versions. Some might perform well out of the box or with minor domain-specific fine-tuning [15]. Generally, the evaluation of LLMs is challenging because of the lack of clarity regarding whether a public benchmark dataset has been used for training. However, when using data from EHRs, it is certain that they have not been used for training a public model.

### Conclusions

NLP has demonstrated the ability to identify and extract cancer-related entities from unstructured medical textual data. Generally, most of the reviewed models showed excellent performance in terms of the *F*_1_-score, and more advanced models outperformed less advanced ones. The BT category performed the best, followed by NN. The use of BTs has increased in recent years. Rule-based applications for IE remain competitive in terms of performance in this specific context.

## Supplementary material

10.2196/68707 Multimedia Appendix 1 Search strategy.

10.2196/68707 Multimedia Appendix 2 Statistical significance of *t* test results.

10.2196/68707 Checklist 1 PRISMA 2020 checklist.

## Abbreviations

BERT: Bidirectional Encoder Representations from Transformers
BT: bidirectional transformer
CRF: conditional random field
EHR: electronic health record
IE: information extraction
LLM: large language model
ML: machine learning
NLP: natural language processing
NN: neural network
PRISMA: Preferred Reporting Items for Systematic Reviews and Meta-Analyses

## References

[1] Evans RS (2016). Electronic health records: then, now, and in the future. Yearb Med Inform.

[2] Ruckdeschel JC, Riley M, Parsatharathy S (2023). Unstructured data are superior to structured data for eliciting quantitative smoking history from the electronic health record. JCO Clin Cancer Inform.

[3] Landolsi MY, Hlaoua L, Ben Romdhane L (2023). Information extraction from electronic medical documents: state of the art and future research directions. Knowl Inf Syst.

[4] Spasić I, Livsey J, Keane JA, Nenadić G (2014). Text mining of cancer-related information: review of current status and future directions. Int J Med Inform.

[5] Hong JC, Fairchild AT, Tanksley JP, Palta M, Tenenbaum JD (2021). Natural language processing for abstraction of cancer treatment toxicities: accuracy versus human experts. JAMIA Open.

[6] Yu S, Le A, Feld E (2021). A natural language processing-assisted extraction system for Gleason scores: development and usability study. JMIR Cancer.

[7] Alkaitis MS, Agrawal MN, Riely GJ, Razavi P, Sontag D (2021). Automated NLP extraction of clinical rationale for treatment discontinuation in breast cancer. JCO Clin Cancer Inform.

[8] Benson R, Winterton C, Winn M (2023). Leveraging natural language processing to extract features of colorectal polyps from pathology reports for epidemiologic study. JCO Clin Cancer Inform.

[9] Si Y, Roberts K (2018). A frame-based NLP system for cancer-related information extraction. AMIA Annu Symp Proc.

[10] AAlAbdulsalam AK, Garvin JH, Redd A, Carter ME, Sweeny C, Meystre SM (2018). Automated extraction and classification of cancer stage mentions from unstructured text fields in a central cancer registry. AMIA Jt Summits Transl Sci Proc.

[11] Osborne JD, O’Leary T, Monte JD, Sasse K, Liang WH (2020). CEUR Workshop Proceedings.

[12] Chen L, Song L, Shao Y, Li D, Ding K (2019). Using natural language processing to extract clinically useful information from Chinese electronic medical records. Int J Med Inform.

[13] Bozkurt S, Magnani CJ, Seneviratne MG, Brooks JD, Hernandez-Boussard T (2022). Expanding the secondary use of prostate cancer real world data: automated classifiers for clinical and pathological stage. Front Digit Health.

[14] Iannantuono GM, Bracken-Clarke D, Floudas CS, Roselli M, Gulley JL, Karzai F (2023). Applications of large language models in cancer care: current evidence and future perspectives. Front Oncol.

[15] Zhou S, Wang N, Wang L, Liu H, Zhang R (2022). CancerBERT: a cancer domain-specific language model for extracting breast cancer phenotypes from electronic health records. J Am Med Inform Assoc.

[16] Fang A, Hu J, Zhao W (2022). Extracting clinical named entity for pituitary adenomas from Chinese electronic medical records. BMC Med Inform Decis Mak.

[17] Zhang X, Zhang Y, Zhang Q (2019). Extracting comprehensive clinical information for breast cancer using deep learning methods. Int J Med Inform.

[18] Kim Y, Meystre SM (2019). A study of medical problem extraction for better disease management. Stud Health Technol Inform.

[19] Coquet J, Bozkurt S, Kan KM (2019). Comparison of orthogonal NLP methods for clinical phenotyping and assessment of bone scan utilization among prostate cancer patients. J Biomed Inform.

[20] Miao S, Xu T, Wu Y (2018). Extraction of BI-RADS findings from breast ultrasound reports in Chinese using deep learning approaches. Int J Med Inform.

[21] Hu D, Liu B, Zhu X, Lu X, Wu N (2024). Zero-shot information extraction from radiological reports using ChatGPT. Int J Med Inform.

[22] Ansoborlo M, Gaborit C, Grammatico-Guillon L, Cuggia M, Bouzille G (2023). Prescreening in oncology trials using medical records. Natural language processing applied on lung cancer multidisciplinary team meeting reports. Health Informatics J.

[23] López-García G, Jerez JM, Ribelles N, Alba E, Veredas FJ (2021). Advances in Computational Intelligence.

[24] Liu H, Zhang Z, Xu Y (2021). Use of BERT (Bidirectional Encoder Representations from Transformers)-based deep learning method for extracting evidences in chinese radiology reports: development of a computer-aided liver cancer diagnosis framework. J Med Internet Res.

[25] Solarte Pabón O, Montenegro O, Torrente M, Rodríguez González A, Provencio M, Menasalvas E (2022). Negation and uncertainty detection in clinical texts written in Spanish: a deep learning-based approach. PeerJ Comput Sci.

[26] Yu Z, Yang X, Dang C (2021). A study of social and behavioral determinants of health in lung cancer patients using transformers-based natural language processing models. AMIA Annu Symp Proc.

[27] Lu Z, Sim JA, Wang JX (2021). Natural language processing and machine learning methods to characterize unstructured patient-reported outcomes: validation study. J Med Internet Res.

[28] Hu D, Zhang H, Li S, Wang Y, Wu N, Lu X (2021). Automatic extraction of lung cancer staging information from computed tomography reports: deep learning approach. JMIR Med Inform.

[29] Hu D, Li S, Zhang H, Wu N, Lu X (2022). Using natural language processing and machine learning to preoperatively predict lymph node metastasis for non-small cell lung cancer with electronic medical records: development and validation study. JMIR Med Inform.

[30] Zitu MM, Zhang S, Owen DH, Chiang C, Li L (2023). Generalizability of machine learning methods in detecting adverse drug events from clinical narratives in electronic medical records. Front Pharmacol.

[31] Seong D, Choi YH, Shin SY, Yi BK (2023). Deep learning approach to detection of colonoscopic information from unstructured reports. BMC Med Inform Decis Mak.

[32] Rohanian O, Jauncey H, Nouriborji M (2023). Proceedings of the 22nd Workshop on Biomedical Natural Language Processing and BioNLP Shared Tasks.

[33] Martín-Noguerol T, López-Úbeda P, Pons-Escoda A, Luna A (2024). Natural language processing deep learning models for the differential between high-grade gliomas and metastasis: what if the key is how we report them?. Eur Radiol.

[34] Thompson J, Hu J, Mudaranthakam DP (2019). Relevant word order vectorization for improved natural language processing in electronic health records. Sci Rep.

[35] Qiu JX, Yoon HJ, Fearn PA, Tourassi GD (2018). Deep learning for automated extraction of primary sites from cancer pathology reports. IEEE J Biomed Health Inform.

[36] Alawad M, Yoon HJ, Tourassi GD Coarse-to-fine multi-task training of convolutional neural networks for automated information extraction from cancer pathology reports.

[37] Dubey AK, Yoon HJ, Tourassi GD Inverse regression for extraction of tumor site from cancer pathology reports.

[38] Wu J, Tang K, Zhang H, Wang C, Li C Structured information extraction of pathology reports with attention-based graph convolutional network.

[39] Odisho AY, Park B, Altieri N (2020). Natural language processing systems for pathology parsing in limited data environments with uncertainty estimation. JAMIA Open.

[40] Alawad M, Gao S, Qiu JX (2020). Automatic extraction of cancer registry reportable information from free-text pathology reports using multitask convolutional neural networks. J Am Med Inform Assoc.

[41] Wu J, Zhang R, Gong T, Liu Y, Wang C, Li C BioIE: biomedical information extraction with multi-head attention enhanced graph convolutional network.

[42] Rios A, Durbin EB, Hands I, Kavuluru R (2021). Assigning ICD-o-3 codes to pathology reports using neural multi-task training with hierarchical regularization.

[43] Park B, Altieri N, DeNero J, Odisho AY, Yu B (2021). Improving natural language information extraction from cancer pathology reports using transfer learning and zero-shot string similarity. JAMIA Open.

[44] Vaswani A, Shazeer N, Parmar N (2017). Attention is all you need. arXiv.

[45] Devlin J, Chang MW, Lee K, Toutanova K (2018). BERT: pre-training of deep bidirectional transformers for language understanding. arXiv.

[46] Kreimeyer K, Foster M, Pandey A (2017). Natural language processing systems for capturing and standardizing unstructured clinical information: a systematic review. J Biomed Inform.

[47] Datta S, Bernstam EV, Roberts K (2019). A frame semantic overview of NLP-based information extraction for cancer-related EHR notes. J Biomed Inform.

[48] Bilal M, Hamza A, Malik N (2025). NLP for analyzing electronic health records and clinical notes in cancer research: a review. J Pain Symptom Manage.

[49] Giorgia T, Johannes CS, Gerasimos S (2021). A study of BERT’s processing of negations to determine sentiment.

[50] Ettinger A (2020). What BERT is not: lessons from a new suite of psycholinguistic diagnostics for language models. Trans Assoc Comput Linguist.

[51] Choi HS, Song JY, Shin KH, Chang JH, Jang BS (2023). Developing prompts from large language model for extracting clinical information from pathology and ultrasound reports in breast cancer. Radiat Oncol J.

