# Supplementary material for: Performance of Natural Language Processing for Information Extraction From Electronic Health Records Within Cancer: Systematic Review
Source: JMIR Med Inform. 2025 Sep 12;13:e68707. doi: 10.2196/68707 (PMC12431712; doi:10.2196/68707)
Supplement: Multimedia Appendix 1 [file medinform-v13-e68707-s001.docx]

**Appendix 1 - Search Strategy**

Pubmed:

*("information extraction"[Title/Abstract] OR "natural language processing"[Title/Abstract] OR nlp[Title/Abstract])*

*AND*

*("EHR"[Title/Abstract] OR "notes"[Title/Abstract] OR "reports"[Title/Abstract])*

*AND*

*(cancer[Title/Abstract] OR tumor[Title/Abstract] OR oncology[Title/Abstract])*

Scopus:

TITLE-ABS(("information extraction" OR "natural language processing" OR nlp) AND (ehr OR notes OR reports) AND (cancer OR tumor OR oncology))

Web of Science

*TI=(("information extraction" OR "natural language processing" OR "NLP") AND (EHR OR notes OR reports) AND (cancer OR tumor OR oncology)) OR*

*AB=(("information extraction" OR "natural language processing" OR "NLP") AND (EHR OR notes OR reports) AND (cancer OR tumor OR oncology))*
