# Supplementary material for: Performance of Natural Language Processing for Information Extraction From Electronic Health Records Within Cancer: Systematic Review
Source: JMIR Med Inform. 2025 Sep 12;13:e68707. doi: 10.2196/68707 (PMC12431712; doi:10.2196/68707)
Supplement: Multimedia Appendix 2 [file medinform-v13-e68707-s002.docx]

**Appendix 2 – Statistical Significance T-Test Results**

|  | Rule-based | Traditional Machine Learning | CRF-based | Neural Network | Bidirectional transformer |
| --- | --- | --- | --- | --- | --- |
| Rule-based |  | 0.0339 | 0.1316 | 0.0907 | 0.1208 |
| Traditional Machine Learning | 0.0339 |  |  | 0.3694 | 0.4719 |
| CRF-based | 0.1316 |  |  | 0.3909 | 0.0873 |
| Neural Network | 0.0907 | 0.3694 | 0.3909 |  | 0.1581 |
| Bidirectional transformer | 0.1208 | 0.4719 | 0.0873 | 0.1581 |  |

Table A1 shows P-values from t-tests comparing F1 differences between model categories. Values ≤ 0.05 indicate statistically significant differences.
